# Diagnostic Value of Endoscopic Ultrasound‐Guided Tissue Acquisition With Rapid On‐Site Evaluation in Small Gastric Subepithelial Lesions

**DOI:** 10.1002/deo2.70282

**Published:** 2026-01-13

**Authors:** Yuki Ikeda, Masahiro Yoshida, Kei Yane, Mayu Shimizu, Keita Seto, Koki Yoshida, Sota Hirokawa, Kotaro Morita, Tetsuya Sumiyoshi, Michiaki Hirayama, Hitoshi Kondo, Kohichi Takada

**Affiliations:** ^1^ Department of Gastroenterology Tonan Hospital Hokkaido Japan; ^2^ Division of Medical Oncology, Department of Internal Medicine Sapporo Medical University School of Medicine Hokkaido Japan

**Keywords:** endoscopic ultrasonography, endoscopic ultrasound‐guided tissue acquisition, gastric subepithelial lesion, gastrointestinal stromal tumor, rapid on‐site evaluation

## Abstract

**Background:**

Gastric subepithelial lesions (SELs) measuring <20 mm without high‐risk features are typically managed with periodic surveillance, while surgical resection is recommended for gastrointestinal stromal tumors (GISTs) per the Japanese guideline. Recent advancements in endoscopic ultrasound‐guided tissue acquisition (EUS‐TA) needles have improved tissue collection, but few studies have assessed the utility of EUS‐TA for SELs <20 mm. This study aimed to evaluate the usefulness of EUS‐TA for gastric SELs <20 mm.

**Methods:**

We retrospectively analyzed patients who underwent EUS‐TA for SELs at Tonan Hospital between June 2012 and March 2025. Variables including needle type, number of passes, histological diagnosis, and diagnostic accuracy were compared between SELs <20 and ≥20 mm. Rapid On‐Site Evaluation (ROSE) was performed for all specimens.

**Results:**

A total of 163 patients were included: 50 with SELs <20 mm and 113 with SELs ≥20 mm. Median lesion size was 15.5 and 31.7 mm, respectively. The <20 mm group required more passes to obtain adequate samples (2.5 vs. 2.0, *p* = 0.03). GIST was the most common diagnosis in both groups, with no significant difference (56% vs. 61.9%). Fine needle biopsy did not significantly improve sample adequacy or diagnostic accuracy. Diagnostic accuracy for SELs <20 mm was comparable to that for SELs ≥20 mm (88% vs. 93.8%).

**Conclusions:**

EUS‐TA with ROSE for gastric SELs <20 mm yields diagnostic accuracy comparable to that for SELs ≥20 mm. Given the high GIST prevalence in small SELs, EUS‐TA may be a valuable diagnostic strategy.

## 1 Introduction

Subepithelial lesions (SELs) are a group of lesions that originate beneath the surface epithelium of the gastrointestinal (GI) tract. They are often asymptomatic and are commonly detected incidentally during GI endoscopy. SELs can arise from various layers of the GI wall and include lesions such as GI stromal tumor (GIST), leiomyoma, lipoma, schwannoma, ectopic pancreas, lymphoma, and others. Endoscopic ultrasound (EUS), typically performed as a subsequent examination to GI endoscopy, is one of the most crucial imaging modalities for evaluating SELs. It enables detailed visualization of the lesion characteristics, including the layer of origin and size, as well as the identification of high‐risk features such as irregular borders, cystic spaces, ulceration, echogenic foci, and heterogeneity [1, 2, 3].

European Society of Gastrointestinal Endoscopy [4] and Japanese [5] guidelines recommend EUS‐guided tissue acquisition (EUS‐TA), including EUS‐guided fine needle aspiration biopsy (EUS‐FNAB) and EUS‐guided core needle biopsy (EUS‐CNB). Such guidelines state that all SELs with features suggestive of GIST, if they are of size greater than 20 mm, with high‐risk features, should provide tissue diagnosis. Gastric SELs <20 mm without high‐risk features should be monitored on GI endoscopy or EUS.

With advancements in EUS‐TA needles, it has become possible to collect a large amount of tissue samples [6, 7]. A meta‐analysis has shown that FNB needles provide higher rates of adequate tissue sampling, better histologic core quality, and improved diagnostic accuracy rate [8]. Moreover, diagnostic accuracy was significantly higher for FNB needles compared to FNA needles, with fewer needle passes required [7, 8, 9]. However, few studies have specifically evaluated the effectiveness of EUS‐TA for SELs <20 mm, leaving the surveillance and management of incidentally detected small SELs controversial. Therefore, the aim of this study was to compare the diagnostic performance of EUS‐TA in gastric SELs smaller than 20 mm with that of SELs ≥ 20 mm.

## 2 Methods

### 2.1 Study Design

The analysis included patients who underwent EUS‐TA for gastric SELs between June 2012 and March 2025. All consecutive patients aged 18 years or over were identified from an endosonography database at Tonan Hospital. This was a retrospective, single‐center study that conformed to the ethics standards of the Declaration of Helsinki and was approved by the Institutional Review Board of Tonan Hospital (a1‐3‐1).

### 2.2 EUS‐TA Procedure

Each EUS‐TA was performed by two expert endosonographers (>100 EUS‐TA procedures) and a few trainees. An oblique‐viewing EUS scope (EG‐740UT or EG‐580UT; FUJIFILM, Tokyo, Japan) was used in all procedures under the supervision of an expert endosonographer. If a trainee failed, a subsequent procedure was performed by an expert endoscopist. All patients were under conscious sedation using intravenous medication and analgesics during the procedure. As FNA needles, Expect (Boston Scientific Japan, Tokyo, Japan), EZShot (Olympus Optical Corp, Tokyo, Japan), and Sonotip (Medicos Hirata, Osaka, Japan) were selected by the endosonographers; Expect and Sonotip were lancet‐type needles, whereas EZShot was a meghini‐type needle. Since the introduction of FNB needles, Acquire (Boston Scientific Japan), Trident (Micro‐Tech, Nanjing, China), and SharkCore (Covidien, Tokyo, Japan) needles were mainly used to obtain larger histological specimens with preserved tissue architecture; Acquire, Trident, and SharkCore were franseen, three‐prong asymmetric, a fork‐tip type needles, respectively.

**FIGURE 1:**
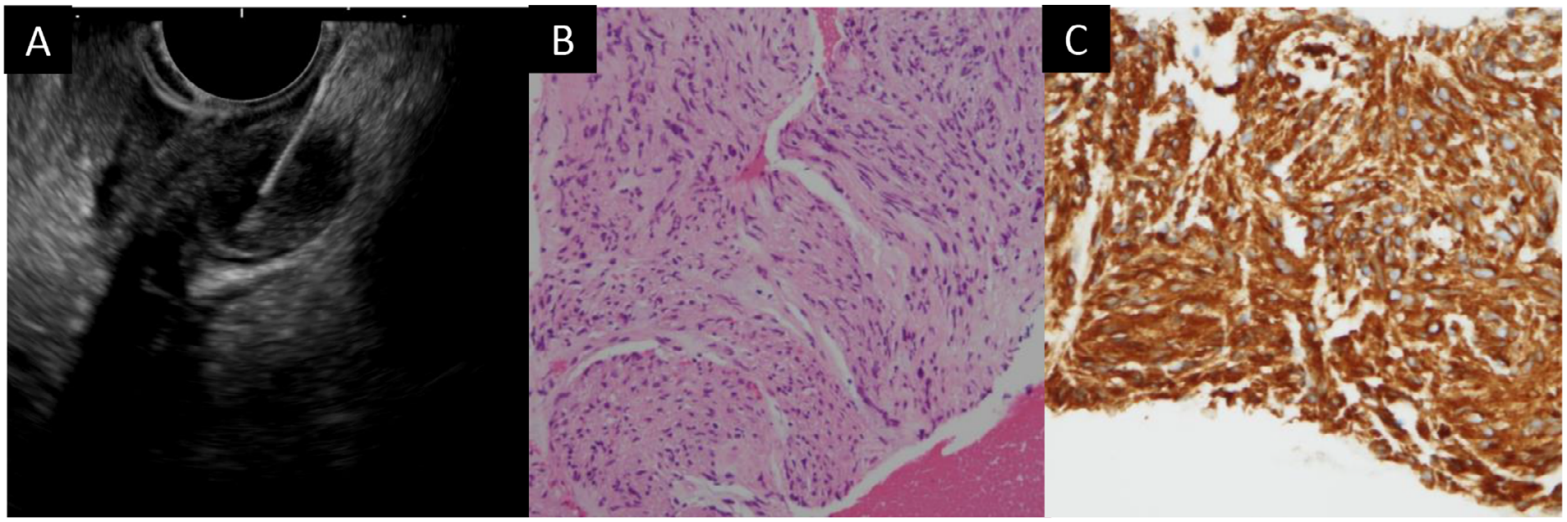
A case of small gastric gastrointestinal stromal tumor (GIST). (A) On endoscopic ultrasound (EUS), a 1.0 cm hypoechoic lesion was identified in the gastric subepithelial layer, and EUS‐guided tissue acquisition (EUS‐TA) was subsequently performed with an EUS‐guided fine needle biopsy (EUS‐FNB) needle (Trident 22G). (B) Histopathological findings of tissue specimens during EUS‐TA revealed spindle tumor cells. (C) GIST was diagnosed based on positive c‐kit immunohistochemical staining.

After an SEL was detected, an FNA or FNB needle was used to obtain tissue specimens under endosonographic guidance for histological diagnosis by endosonographers. Puncturing the SEL with an FNA or FNB needle under EUS guidance, the stylet was removed, and approximately 15 to‐and‐fro movements were performed using a syringe with 20 mL negative pressure (defined as the dry‐suction technique) attached to the needle (Figure 1). If this technique yielded a blood specimen, a slow‐pull technique, consisting of gradually withdrawing the stylet, was performed. Once the tissue specimen was collected, rapid on‐site evaluation (ROSE) was performed under a microscope by a cytopathologist or cytotechnologist. Additional passes were allowed at the endosonographers’ discretion until a cytopathologist or cytotechnologist deemed the obtained specimen to be adequate.

### 2.3 Histological Assessment

Tissue specimens from an EUS‐TA procedure were fixed in 10% formalin. Tissue blocks were then embedded in paraffin for histopathological evaluation after hematoxylin and eosin, and immunohistochemistry (IHC) staining. A GIST was diagnosed based on the presence of spindle or epithelioid cell tumors, and subsequent positive c‐kit and/or CD34 IHC staining. Leiomyomas were diagnosed after positive desmin staining, and schwannomas after positive S‐100 staining. An ectopic pancreas and lipomas were not necessarily required for a diagnosis.

### 2.4 Outcomes

The final diagnosis was made by the histopathological evaluation of resected specimens from patients who underwent surgery for SELs. If surgical resection was not performed due to the presence of benign disease, periodic GI endoscopy or EUS surveillance as clinical follow‐up (median, 50 months; range, 4–102 months) was monitored without treatment. The pathological evaluation of benign disease based on EUS‐TA was considered the final diagnosis in the absence of changes in the lesion. Among patients with SELs <20 mm who underwent surgical resection based on a preoperative diagnosis of GIST, postoperative pathological findings were reviewed, and recurrence risk stratification according to the modified Fletcher/Joensuu classification [10] was assessed. The primary outcome was to compare the adequacy of the specimen collection rate for histopathological assessment between the two groups, defined as the capability of IHC staining when required. Secondary outcomes included needle types, numbers of passes, frequency of GIST, diagnostic accuracy, and recurrence risk stratification in patients with surgically resected GISTs <20 mm.

### 2.5 Statistical Analysis

As a Chi‐square test, Fisher's exact test was used to compare categorical variables. A Mann–Whitney *U*‐test was used for nonparametric continuous variables. A two‐sided *p*‐value of <0.05 was considered statistically significant. All statistical analyses were performed using EZR software (Saitama Medical Center, Jichi Medical University, Saitama, Japan).

## 3 Results

### 3.1 Patients’ Characteristics

**FIGURE 2:**
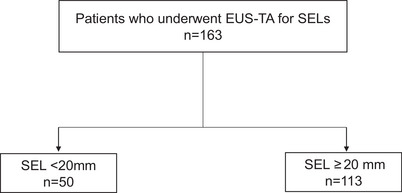
Flow chart of this study design.

From June 2012 to March 2025, a total of 163 patients who underwent EUS‐TA procedures for gastric SELs were included in this study: 50 patients were assigned to the SELs measuring <20 mm group and 113 patients to the SELs ≥20 mm group (Figure 2). The median age was 69 years (range 35–83 years) with 33 females (66%) in the <20 mm group, and 64 years (range 33–90 years) with 53 females (46.9%) in the ≥20 mm group. The median size of a lesion was 15.5 mm in the <20 mm group, and 31.7 mm in the ≥20 mm group, respectively. In the <20 mm group, lesion enlargement was noted in 24 patients (48%). Patients’ characteristics, including features of SELs, are shown in Table 1.

**TABLE 1:** Patients’ characteristics according to endoscopic ultrasound‐guided tissue acquisition (EUS‐TA).

	<20 mm (*n* = 50)	≥20 mm (*n* = 113)	*p*‐value
Age, years (range)	69 (35–83)	64 (33–90)	0.18
Gender			
Male, *n* (%)	17 (34)	60 (53.1)	0.03
Female, *n* (%)	33 (66)	53 (46.9)	
Location of SEL, *n* (%)			
upper part	28 (56)	61 (54)	0.58
middle part	13 (26)	37 (32.7)	
lower part	9 (18)	15 (13.3)	
High‐risk GI endoscopy features, *n* (%)	24 (48)	22 (19.4)	< 0.001
irregular border	0	10 (8.8)	
ulceration	0	5 (4.4)	
enlargement	24 (48)	7 (6.2)	
Lesion size, mm (range)	15.5 (8–19)	31.7 (20–190)	<0.001

Abbreviations: EUS‐TA, endoscopic ultrasound‐guided tissue acquisition; GI, gastrointestinal; NS, not significant; SEL, subepithelial lesion.

Data are expressed as median (range) or *N* (%).

### 3.2 Comparison of EUS‐TA Procedures

A comparison of EUS‐TA procedures is shown in Table 2. No significant difference was noted in the proportional use of FNA and FNB needles. The most frequent needle used in the <20 mm group was the Lancet needle 22G (46%), and the Franseen needle 22G (54.9%) in the ≥ 20 mm group. The median number of passes was significantly higher (2.5 vs. 2.0, *p* = 0.03). Only two patients of the ≥20 mm group showed bleeding after procedures. One case developed a perilesional hematoma that resolved spontaneously, while another case experienced puncture‐site bleeding requiring endoscopic clipping for hemostasis.

**TABLE 2:** Comparison of procedures for gastric SELs <20 mm and those ≥20 mm.

	<20 mm (*n* = 50)	≥20 mm (*n* = 113)	*p*‐value
FNA needle/FNB needle, *n* (%)	23 (46)/27 (54)	48 (42.4)/65 (57.5)	0.17
FNA needle			
Lancet type 22G	23 (46)	39 (34.5)	
Lancet type 19G	0	6 (5.3)	
Menghini tip 22G	0	3 (2.7)	
FNB needle			
Franseen type 22G	22 (44)	62 (54.9)	
Three‐prong asymmetric type 22G	4 (8)	3 (2.7)	
Fork‐tip type 22G	1 (2)	0	
Aspiration techniques			
Dry‐suction	44 (88)	82 (72.6)	
Slow‐pull	3 (6)	7 (6.2)	
Both dry‐suction and slow‐pull	2 (4)	19 (16.8)	
Unknown	1 (2)	5 (4.4)	
The number of passes (range)	2.5 (1–6)	2 (1–6)	0.03

Abbreviations: EUS‐TA, endoscopic ultrasound‐guided tissue acquisition; FNA, fine needle aspiration; FNB, fine needle biopsy; NS, not significant; SELs, subepithelial lesions.

Data are expressed as median (range) or *N* (%).

### 3.3 Diagnostic Yield of EUS‐TA

Of the 50 patients in the SELs <20 mm group, the most frequently observed diagnosis was GIST, found in 28 patients (56%), followed by ectopic pancreas in six patients (12%), leiomyoma in five patients (10%), schwannoma in two patients (4%), and B‐cell lymphoma in two patients (4%). Meanwhile, of the 113 patients in the ≥20 mm group, 70 (61.9%) had a GIST, 16 (14.2%) had a leiomyoma, 9 (8%) had a schwannoma, and six (5.3%) had an ectopic pancreas. GIST was the most common pathological diagnosis in both groups, with no significant difference found (56% vs. 61.9%, *p* = 0.49). Adequate specimens were obtained from 44 patients (88%) in the <20 mm group and 106 patients (93.8%) in the ≥20 mm group. No statistically significant differences in the rate of procuring adequate specimens between the two groups were noted (88% vs. 93.8%, *p* = 0.22; Table 3). The adequate specimen acquisition rate was 92.4% (85/92) for FNB needles and 91.5% (65/71) for FNA needles, showing no statistically significant difference. Similarly, for the <20 mm group, the acquisition rates were 85.2% (23/27) for FNB and 91.3% (21/23) for FNA, also without a significant difference (Table 4). Of the 28 patients diagnosed with GIST <20 mm via EUS‐TA, and one additional patient with suspected GIST despite inconclusive EUS‐TA results, 27 patients underwent surgical resection. The risk classification of surgically resected GIST is shown in Table 5. Very low risk was the most common classification, observed in 20 patients (74.1%), followed by low risk in three patients (11.1%), intermediate risk in one patient (3.7%), and high risk in three patients (11.1%). In the three patients with high‐risk GIST <20 mm, tumor enlargement was observed compared to a previous examination; however, no signs of an irregular border or ulceration were noted.

**TABLE 3:** Outcomes of endoscopic ultrasound‐guided tissue acquisition (EUS‐TA) procedures.

	<20 mm (*n* = 50)	≥20 mm (*n* = 113)	*p*‐value
Adequate specimen, *n* (%)	44 (88)	106 (93.8)	0.22
GIST	28 (56)	70 (61.9)	0.49
Ectopic pancreas	6 (12)	6 (5.3)	
Leiomyoma	5 (10)	16 (14.2)	
Schwannoma	2 (4)	9 (8)	
B‐cell lymphoma	2 (4)	1 (0.9)	
Lipoma	1 (2)	1 (0.9)	
Glomus tumor	0	1 (0.9)	
Adenocarcinoma	0	1 (0.9)	

Abbreviations: EUS‐TA, endoscopic ultrasound‐guided tissue acquisition; GIST, gastrointestinal stromal tumor; NS, not significant.

Data are expressed as *N* (%).

**TABLE 4:** Comparison of adequate specimen collection rates for fine needle aspiration (FNA) and fine needle biopsy (FNB) needles.

	FNA needle	FNB needle	*p*‐value
All lesions	65/71 (91.5)	85/92 (92.4)	1
< 20 mm	21/23 (91.3)	23/27 (85.2)	0.67
≥ 20 mm	44/48 (91.7)	62/65 (95.4)	0.46

Abbreviations: FNA, fine needle aspiration; FNB, fine needle biopsy; NS, not significant.

Data are expressed as median (range) or *N* (%).

**TABLE 5:** Risk classification of surgically resected gastrointestinal stromal tumors (GISTs) <20 mm.

	Surgically resected GIST <20 mm (*n* = 27)	
Risk category (%)	Very low risk	20 (74.1)
	Low risk	3 (11.1)
	Intermediate risk	1 (3.7)
	High risk	3 (11.1)

Abbreviation: GIST, gastrointestinal stromal tumor.

Data are expressed as *N* (%).

## 4 Discussion

Gastric SELs are often asymptomatic and identified incidentally during GI endoscopy, although they might be identified during computed tomography or magnetic resonance imaging. In particular, small SELs do not induce any signs or symptoms in patients despite some having malignant potential. Small gastric SELs present a diagnostic challenge. EUS imaging alone may be insufficient for a definitive diagnosis [11, 12, 13], especially in lesions lacking the typical features of GIST, leiomyoma, or other mesenchymal tumors. Histological confirmation, including immunohistochemical staining, is often essential even for small lesions to guide appropriate management strategies. The addition of TA to EUS‐FNA/FNB showed this to increase diagnostic accuracy compared with EUS without TA [11, 14, 15, 16]. The guidelines of the European Society of Gastrointestinal Endoscopy [4], the National Comprehensive Cancer Network [17], and American College of Gastroenterology [18], and the Japanese guidelines suggest providing a tissue diagnosis (e.g., EUS‐TA) for SELs with features suggestive of GIST, sizes of <20 mm, or with high‐risk features.

Several studies have evaluated the diagnostic performance of EUS‐TA, including EUS‐FNA and EUS‐FNB, in gastric SELs. The reported diagnostic yield and accuracy vary depending on lesion characteristics, needle type, and procedural techniques. A systematic review and meta‐analysis of 94 studies evaluated pooled rates of diagnostic yield and complications in SELs of the upper GI, comparing endoscopic biopsy, EUS‐FNA, EUS‐FNB, and mucosal incision‐assisted biopsy (MIAB). Diagnostic yields were 40.6% for endoscopic biopsy, 74.6% for EUS‐FNA, 84.2% for EUS‐FNB, and 88.2% for MIAB, leading to the conclusion that EUS‐FNB and MIAB were superior to endoscopic biopsy and EUS‐FNA [19]. A meta‐analysis of 10 studies comparing EUS‐FNB sampling performance with FNA in GI SELs demonstrated higher pooled rates of adequate samples for FNB (94.9% vs. 80.6%, *p* = 0.007) and histologic core procurement (89.7% vs. 65%, *p* < 0.0001). Furthermore, FNB was associated with a significantly greater diagnostic accuracy and a smaller number of passes compared to FNA, whereas no significant difference between FNB and FNA was observed when ROSE was available [8].

Several studies on the comparative diagnostic accuracy of endoscopic techniques for small SELs were reported. For small SELs <20 mm, EUS‐FNB using Franseen‐type needles demonstrated 85%–94% diagnostic rate [20, 21], and EUS‐FNB with fork tip needles achieved 84%–89% diagnostic accuracy with size 11–20 mm lesions [22]. A systematic review with network meta‐analysis was made of eight randomized controlled trials evaluating the sample adequacy, diagnostic accuracy, and complications of upper GI SELs. In the subgroup analysis that included SELs <20 mm, MIAB showed superior accuracy compared to FNA, while FNB and FNA were comparable. Although FNB ranked highest for overall diagnostic performance, MIAB was ranked superior in both adequacy and accuracy for SELs <20 mm. When ROSE was used, the difference between EUS‐FNB, EUS‐FNA, and MIAB was diminished [23].

In this study, we evaluated the utility and clinical validity of EUS‐TA combined with ROSE for the diagnosis of small gastric SELs measuring <20 mm. The rate of procuring adequate specimens for SELs <20 mm was comparable to that for SELs ≥20 mm, with no statistically significant difference (88% vs. 93.8%). Moreover, no statistically significant difference in diagnostic accuracy was observed between FNB and FNA, regardless of the size of gastric SELs. Despite a smaller lesion size, adequate sample acquisition was achieved in the majority of cases, although a higher number of needle passes was required for lesions <20 mm (2.5 vs. 2.0, *p* = 0.03). The use of FNB needles did not significantly improve sample adequacy or diagnostic accuracy in our study. This result is attributed to the high diagnostic yield of FNA combined with ROSE in our study, suggesting that the use of ROSE may be beneficial for the diagnosis of SELs regardless of the type of needle used. This is particularly valuable in small lesions, where tissue volume is limited. The real‐time cytopathological assessment facilitated by ROSE likely reduced nondiagnostic rates and increased confidence in the diagnosis. Furthermore, over half (56%) of SELs <20 mm in our study were pathologically confirmed as GIST; these are potentially malignant and may benefit from early therapeutic intervention. These findings suggest that EUS‐TA is a feasible and reliable diagnostic tool, even for small lesions that are traditionally monitored. MIAB showed higher tissue sampling and diagnostic yields for lesions <20 mm compared with EUS‐FNAB [24]; However, this may be attributable to the predominant use of FNA needles or early‐generation FNB needles. With the current availability of new‐generation FNB needles with improved TA capability, outcomes comparable to MIAB may be expected.

The management of pathologically diagnosed GIST <20 mm remains controversial, and remains markedly different between several guidelines. The European Society for Medical Oncology and Japanese guidelines recommend that all histologically diagnosed GISTs should be surgically resected if they are of a size <20 mm [5, 25 ]. Meanwhile, National Comprehensive Cancer Network guidelines suggest that periodic endoscopic or radiologic surveillance should be considered if no high‐risk EUS features exist, including an irregular border, cystic spaces, ulceration, echogenic foci, and heterogeneity. Surgical resection is recommended for gastric GIST <20 mm with high‐risk EUS features or biopsy features such as the presence of mitoses and/or tumor necrosis [18]. Of the 27 patients who underwent surgical resection with SELs <20 mm, three patients (11.1%) were classified into the high‐risk group according to the risk classification (modified Fletcher/Joensuu classification) [10] used in our study. Since no procedure‐related complications or false‐positive diagnoses (non‐GIST on postoperative pathology) were observed in SELs <20 mm, accurate diagnosis and appropriate surgical management of GIST <20 mm are considered clinically meaningful.

Several limitations exist in this study. First, it was a single‐center retrospective analysis, which may limit the generalizability of the findings. Second, the small sample size precluded the determination of optimal lesion size for performing EUS‐TA in gastric SELs. Third, long‐term follow‐up data were not analyzed, so the clinical outcomes of patients with small GISTs remain uncertain. Fourth, selection bias may be present because EUS‐TA for gastric SELs <20 mm was performed based on the endoscopist's discretion —typically when lesion growth was suspected—without predefined criteria. Finally, since ROSE was applied in all cases, we could not directly assess whether the combination of EUS‐FNB and ROSE offers a diagnostic yield compared to EUS‐FNB alone. Future prospective multicenter studies are warranted to validate our findings and to determine whether the use of EUS‐TA with ROSE can provide high diagnostic accuracy of small gastric SELs. Additionally, the development of novel needles may further enhance TA, especially in small SELs.

## 5 Conclusions

In conclusion, EUS‐TA with ROSE appears to be a valuable diagnostic approach for small gastric SELs, offering improved diagnostic yield and safety. This method may be especially beneficial. Given the high proportion of small lesions ultimately diagnosed as GISTs, TA should be considered even for small lesions.

## Author Contributions

**Yuki Ikeda**: conceptualization, data extraction, investigation, studies assessment, and drafting manuscript. **Masahiro Yoshida**: conceptualization, data extraction, investigation, and studies assessment. **Kei Yane**: conceptualization and studies assessment. **Mayu Shimizu**, **Keita Seto**, **Koki Yoshida**, **Sota Hirokawa**, **Kotaro Morita**, **Tetsuya Sumiyoshi**, **Michiaki Hirayama**, **and Hitoshi Kondo**: investigation. **Kohichi Takada**: supervision.

## Conflicts of Interest

The authors declare no conflicts of interest.

## Funding

The authors received no specific funding for this study.

## Consent

Written informed consent was obtained from all patients prior to undergoing EUS‐TA. Patients were given the opportunity to opt out of participation through a notice posted on the hospital's website.

## Ethics Statement

**Approval of the research protocol by an Institutional Reviewer Board (IRB)**: This study was approved by the Ethics Committee of Tonan Hospital (Approval No: a1‐3‐1) and was conducted in accordance with the ethical standards of the Declaration of Helsinki.
